# Stress Field Prediction and DIC-Informed Spatial Regularization for AlSi10Mg Alloy Tensile Specimens Based on Graph Neural Networks

**DOI:** 10.3390/ma19183979

**Published:** 2026-09-19

**Authors:** Zhongying Dong, Xingbo Xie, Guili Yang, Zhangdong Li, Xinghua Li, Huayuan Ma

**Affiliations:** College of Field Engineering, Army Engineering University of PLA, Nanjing 210007, China; dzying123@aeu.edu.cn (Z.D.); znbxie@126.com (X.X.); yangguili2026@163.com (G.Y.); l15709975@163.com (Z.L.)

**Keywords:** AlSi10Mg, graph neural network, full-field stress prediction, digital image correlation, DIC-informed spatial regularization, ablation study

## Abstract

**Highlights:**

- FiLM-GNN predicts von Mises stress with R^2^ = 0.9984 and RMSE = 4.60 MPa.
- The independent force branch reconstructs dense curves with R^2^ = 0.9957.
- DIC-informed spatial regularization improves DIC spatial shape loss by 3.21% with 0.232 MPa mean correction.
- The framework enables rapid full-field prediction with conservative experiment-informed spatial regularization.
- Field–force decoupling enables curve-specific tuning without altering spatial fields.
- Screenshot-derived pseudo-DIC provides relative spatial cues for regularization when raw experimental matrices are unavailable.

**Abstract:**

High-fidelity finite-element (FE) simulation of nonlinear full-field responses in AlSi10Mg tensile specimens is computationally expensive, while the available digital image correlation (DIC) data in this study consist only of engineering-strain screenshots rather than exportable numerical fields. This work therefore develops a simulation-to-experiment framework for rapid full-field stress prediction and weakly supervised DIC-informed spatial regularization. Two hundred independent Abaqus/Explicit loading cases generated 5600 graph samples, which were used to train an eight-layer FiLM-conditioned graph neural network for nodal von Mises stress and three-component displacement prediction, together with a decoupled structured force–displacement branch. On independent FE tests, von Mises stress prediction achieved R^2^ = 0.9984 with an RMSE of 4.60 MPa, while the dense force–displacement curve achieved R^2^ = 0.9957 and RMSE = 59.70 N. Five machine tensile tests gave mean pre-fracture curve NRMSEs of 4.40% for FE versus experiment and 5.31% for GNN versus experiment. On independent specimens L-7 and L-8, the constrained adapter reduced DIC spatial shape loss by 3.21% with a mean absolute stress correction of 0.232 MPa. The framework therefore provides rapid FE-surrogate prediction with conservative experiment-informed spatial regularization rather than experimental stress inversion.

## 1. Introduction

AlSi10Mg is a widely used lightweight Al-Si-Mg alloy whose macroscopic tensile response and local failure behavior are jointly influenced by processing condition, defect distribution, and material variability [1,2,3]. Uniaxial tensile tests readily provide force–displacement or stress–strain curves, but they do not directly reveal the spatiotemporal evolution of the surface stress field. Finite-element methods can resolve nodal stress, displacement, plastic deformation, and damage; however, nonlinear constitutive behavior, element deletion, and multi-case parametric analyses substantially increase computational cost, limiting their use in rapid assessment, online analysis, and parameter inversion [4,5].

Data-driven surrogate models reduce the repeated cost of high-fidelity simulations. Graph neural networks operate directly on FE node-edge topology and have demonstrated their effectiveness for mesh-based stress, strain, and displacement-field prediction [4,5,6,7], but simulation-trained mechanical-field surrogates can remain sensitive to numerical–experimental discrepancies.

Digital image correlation provides non-contact, full-field surface displacement and strain measurements [8,9], but the present archive contains only engineering-strain screenshots rather than numerical DIC matrices. The screenshots are therefore used to recover relative spatial patterns that constrain the spatial ordering and gradient morphology of the predicted stress field without attempting absolute stress inversion.

Conventional inverse analysis and reduced-order approaches such as POD/POD-RBF can identify material parameters or reconstruct mechanical fields efficiently when quantitative experimental observables and a parameterized reduced basis are available [10,11]. In contrast, the present workflow combines a fixed-topology FE surrogate with the conservative fusion of relative screenshot-derived spatial information and is not intended to replace classical inverse identification.

**Figure 1:**
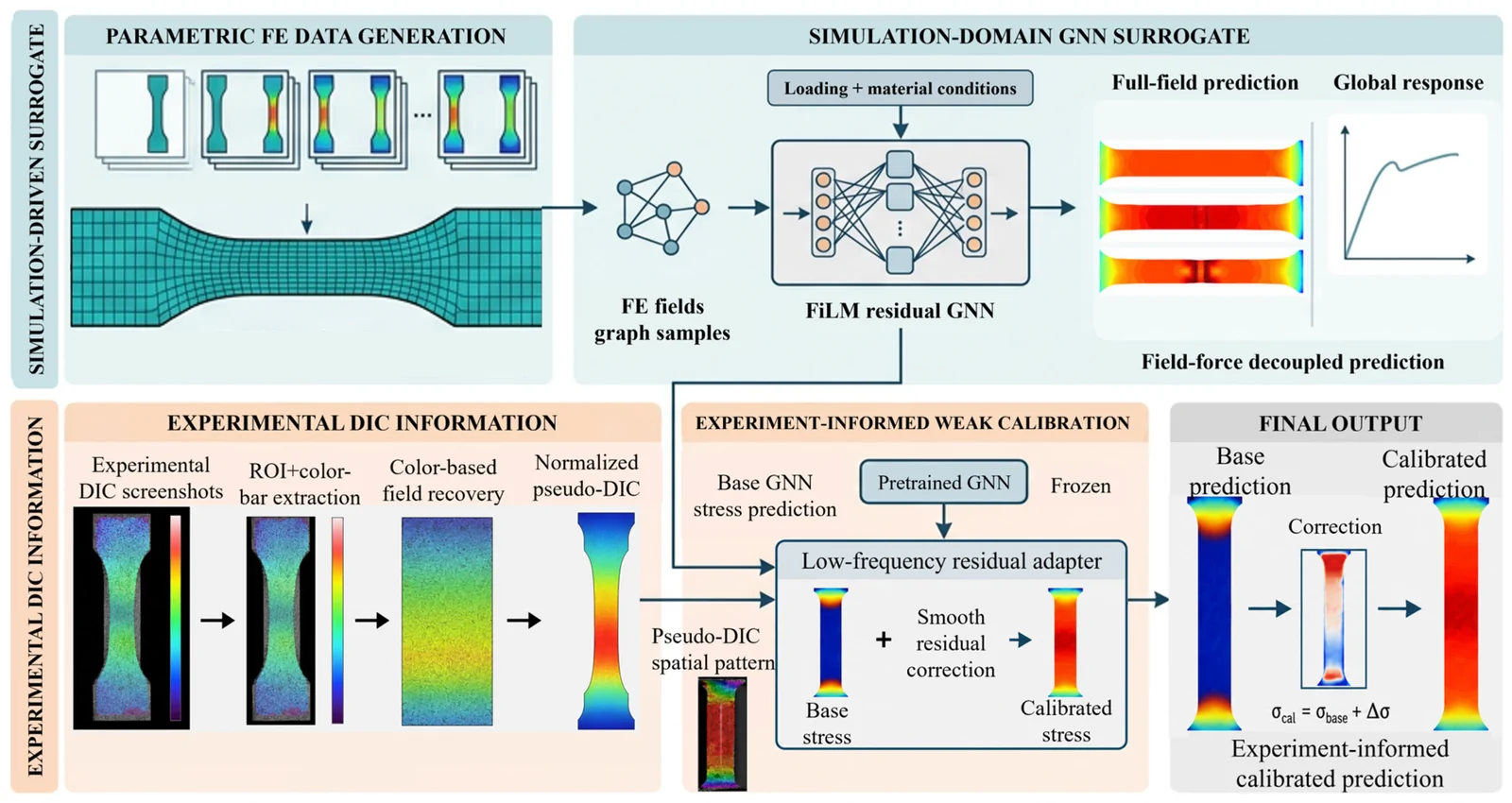
Overall workflow of the proposed framework.

This study establishes an end-to-end workflow (Figure 1) spanning parametric explicit FE analysis, graph-structured data extraction, field-force decoupled GNN pretraining, DIC screenshot reconstruction, and weakly supervised experiment-informed spatial regularization. The main contributions are fourfold: (1) a three-dimensional AlSi10Mg tensile graph dataset incorporating elastoplasticity, damage, element deletion, and dense force–displacement histories; (2) an eight-layer FiLM-conditioned displacement-query field predictor with a structured force branch decoupled from the graph backbone; (3) a DIC screenshot-processing pipeline for frame-wise color bar reconstruction, CIELAB color matching, contour completion, confidence estimation, and pseudo-DIC field construction; and (4) a low-frequency residual adapter trained with the base model frozen for experiment-informed spatial regularization of the simulation-derived stress field, evaluated through independent-specimen testing, controlled ablations, and training specimen analysis.

## 2. Construction of the Parametric Finite-Element Graph Dataset

### 2.1. Specimen Geometry, Material Model, and Finite-Element Setup

A full three-dimensional AlSi10Mg tensile specimen consistent with the experimental geometry was modeled. The total specimen length was 80.94 mm; the gauge section was 6 mm wide and 32 mm long; the grip width was 10 mm; and the thickness was 2 mm. No symmetry reduction was applied so that the complete surface topology was retained for registration with the DIC observation region. FE analyses were performed in Abaqus/Explicit 2024 (Dassault Systèmes, Vélizy-Villacoublay, France) using primarily C3D8R solid elements with an overall mesh size of approximately 0.5 mm. One end of the specimen was fixed, and a maximum axial displacement of 6.0 mm was prescribed at the opposite end. All parametric cases used the same geometry, mesh, and boundary conditions. The principal material and FE settings are summarized in Table 1. The mechanical behavior of AlSi10Mg is strongly affected by heat-treatment condition, and previous studies have shown that T6 and rapid heat treatments can substantially modify its microstructure and strength–ductility balance [12,13,14,15].

The constitutive response uses isotropic J2 (von Mises) elastoplasticity with E = 60 GPa, ν = 0.30, and tabulated multilinear isotropic hardening from 133.99 MPa at zero plastic strain to 415 MPa at a plastic strain of 0.38. Ductile damage initiation is specified at stress triaxiality 0.33 and strain rate 0. The bulk region uses an initiation strain of 0.35 and displacement-based damage evolution of 0.001 mm, whereas the approximately 1 mm central weakened band (y = −15.47 ± 0.50 mm) retains the same elastic–plastic response but uses an initiation strain of 0.14 and damage evolution displacement of 0.15 mm. Element deletion is enabled after damage evolution, and semi-automatic mass scaling targets a stable time increment of 1 × 10^−6^ s. Abaqus/Explicit was selected to accommodate the combined elastoplasticity, damage evolution, and element deletion. Kinematic couplings connect the specimen ends to reference points; one reference point is fully constrained and the opposite reference point receives a smooth-step axial displacement reaching 6.0 mm over 0.06 s (nominal rate 100 mm/s).

Two auxiliary numerical checks were performed separately from the 200-case learning dataset. A representative nominal analysis using the production settings recorded whole-model ALLKE and ALLIE histories to verify quasi-static behavior, and a three-level mesh sensitivity study used global seeds of 0.50, 0.40, and 0.35 mm while keeping the geometry, constitutive parameters, weakened-region definition, loading history, mass scaling, and boundary conditions unchanged.

### 2.2. Elastic Modulus Perturbation and LHS Case Design

To represent stiffness variability under a fixed geometry, the elastic modulus ratio was selected as the sampling variable. Latin hypercube sampling was performed over the interval 0.92–1.08 to generate 199 perturbed cases, and one nominal case was added to obtain 200 independent FE loading trajectories. A maximin sampling criterion was used to improve the uniformity of coverage within the interval [16]. Because only the elastic modulus was varied in the present dataset, the simulation-domain generalization claims are restricted to this single-parameter range and are not extrapolated to joint uncertainty in yield, hardening, or damage parameters. Across this dataset, geometry, mesh topology, boundary conditions, and the prescribed central weakened region are fixed; therefore, the held-out accuracy represents interpolation within this restricted single-parameter domain rather than broad physical generalization.

### 2.3. Batch Simulation, Failure Identification, and Field Extraction

The Abaqus Python interface was used to automate parametric model generation, solution, and result extraction. For each case, 28 aligned field frames were selected over the 0–5.99 mm loading range, with denser sampling near necking and the terminal damage stage. A high-resolution reaction force displacement history was recorded in parallel to balance the size of the full-field dataset against the fidelity of the global response description. Nodal coordinates, displacement, von Mises stress, plastic strain, reaction force, and damage-state variables were extracted from the ODB files. Nodal fields were obtained by averaging element nodal results at shared nodes, with nodal von Mises stress and three-component displacement used as the primary supervised targets. Reaction force histories were resampled onto a common displacement grid to obtain a complete force–displacement curve for each case. The terminal failure state was identified from a combination of element deletion status and reaction force drop, and dynamic residual forces after failure were removed. The 200 cases ultimately yielded 5600 graph samples, each containing the shared topology, loading conditions, nodal field labels, and the corresponding dense force–displacement history. Similar multi-case crystal-plasticity FE analyses were used to investigate residual stress and localized deformation in additively manufactured components [17]. The central weakened band is a prescribed FE prior, so the workflow does not constitute prediction of an arbitrary crack-initiation location or path.

### 2.4. Graph Representation, Input Features, and Dataset Split

All cases share the same FE topology. FE nodes are mapped to graph nodes, while solid-element connectivity is expanded into bidirectional graph edges. The topology is reused during training and inference; only the loading conditions and case-dependent labels are updated. The resulting shared graph contains 17,325 nodes and 47,580 directed edges, and the external surface is represented by 16,064 triangular facets. Each node has a 32-dimensional input comprising normalized coordinates, boundary condition indicators, node degree, and Fourier encodings of the coordinates. Each edge has four features describing the three-dimensional relative position and Euclidean distance between neighboring nodes. The model follows a displacement-query formulation and therefore does not depend on the prediction from the previous loading state; nodal fields can be queried directly at any prescribed displacement. The 14-dimensional global condition vector contains the normalized displacement, Fourier encoding of the displacement, and material conditions. Supervision includes nodal von Mises stress, three-component displacement, a graph-level guide force, and the dense force–displacement curve. The dataset is split by complete FE cases into 183 training cases, 8 validation cases, and 9 test cases, corresponding to 5124, 224, and 252 field samples, respectively. All loading frames from a given case remain within a single subset to prevent leakage caused by correlations between adjacent states. The relational inductive bias of graph networks makes them well suited to physical-field learning on irregular discretizations [18]. All supervised field labels are numerical nodal arrays extracted directly from Abaqus ODB field/history outputs; rendered FE contour images are not used as GNN inputs or labels.

## 3. Field-Force Decoupled Graph Neural Network

### 3.1. Overall Architecture

The model consists of a graph-based field prediction backbone and an independent force–displacement branch. The graph backbone predicts nodal von Mises stress and displacement from node features, edge features, and displacement material conditions, whereas the independent force branch predicts the global loading force using only displacement and material conditions. The two functions are decoupled so that the spatial fields and the global response curve can be optimized separately. The overall mapping is given in Equation (1).

(1) $$\begin{array}{l} \left(\hat{\sigma },\hat{U}\right)=f_{\theta }\left(G,X,E_{f},c\right)\\ \hat{F}\left(\tilde{\delta }\right)=g_{\phi }\left(\tilde{\delta },\tilde{m}\right) \end{array}$$

where $f_{\theta }$ denotes the graph-based field prediction network parameterized by the trainable parameters θ; $\hat{\sigma }$ and $\hat{U}$ denote the predicted nodal von Mises stress and three-component displacement, respectively. G represents the finite-element graph topology, X the node-feature matrix, $E_{f}$ the edge-feature matrix, and c the global displacement–material conditioning vector. $g_{\phi }$ denotes the independent structured force–displacement branch with trainable parameters ϕ. $\tilde{\delta }$ and $\tilde{m}$ denote the normalized loading displacement and normalized material-parameter vector, respectively.

**Figure 2:**
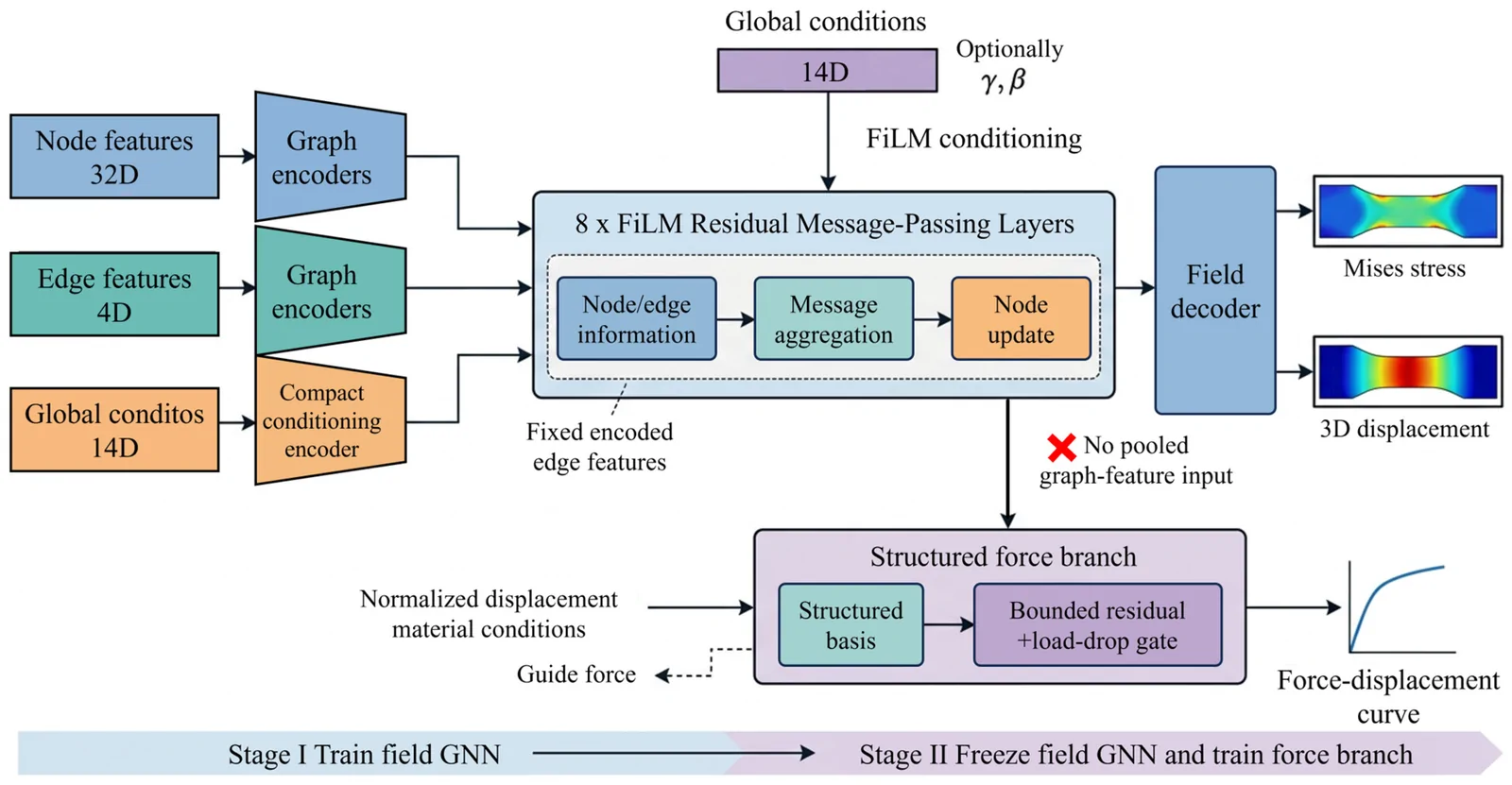
Architecture of the field-force decoupled GNN.

The graph backbone comprises node and edge encoders, a condition encoder, an eight-layer FiLM message-passing processor, and a field decoder (Figure 2). The independent structured branch is used to reconstruct the complete force–displacement response [4,19]. Sanchez-Gonzalez et al. [19] demonstrated the extrapolation capability of graph networks in long-horizon simulations of complex physical systems, providing an architectural basis for the displacement-query field predictor adopted here. In parallel, decoupled neural network formulations have been shown to separate local field features from global response trends in real-time structural fatigue prediction [20].

### 3.2. Node, Edge, and Condition Encoding

Node, edge, and condition inputs are independently mapped into latent spaces using multilayer perceptrons. The condition encoder produces a displacement-material embedding that generates FiLM scale and shift parameters at each message-passing layer, allowing feature propagation over the shared mesh to adapt to the loading stage and material condition.

FiLM modulation is defined in Equation (2). The channel-wise scale and shift terms are generated from the condition embedding, enabling loading condition injection without modifying the graph topology.

(2) $${\mathrm{FiLM}}^{\left(l\right)}\left(\Delta h,z_{c}\right)=\left[1+\gamma^{\left(l\right)}\left(z_{c}\right)\right]⊙\Delta h+\beta^{\left(l\right)}\left(z_{c}\right)$$

In Equation (2), γ and β denote the layer-specific scale and shift generated from the condition embedding, and ⊙ denotes element-wise multiplication. The multiplicative scale is written in residual form as 1 + γ so that the network remains closed to the unmodulated state at the beginning of training, thereby reducing abrupt feature perturbations caused by condition injection.

### 3.3. Eight-Layer Conditional Residual Message Passing

Message construction, summation aggregation, and conditionally modulated residual updating constitute the core operations of one message-passing layer, as expressed in Equation (3). The encoded edge hidden states remain fixed throughout the processor, while the node states sequentially integrate local geometry and loading conditions through eight message-passing layers.

(3) $$\begin{array}{l} m_{ij}^{\left(l\right)}=\phi_{e}^{\left(l\right)}\left(h_{i}^{\left(l\right)}\|h_{j}^{\left(l\right)}\|h_{ij}^{e}\right)\\ {\bar{m}}_{j}^{\left(l\right)}=\sum_{i\in N(j)}m_{ij}^{\left(l\right)}\\ h_{j}^{\left(l+1\right)}=h_{j}^{\left(l\right)}+{\mathrm{FiLM}}^{\left(l\right)}\left[\phi_{v}^{\left(l\right)}\left(h_{j}^{\left(l\right)}\|{\bar{m}}_{j}^{\left(l\right)}\right),z_{c}\right] \end{array}$$

In Equation (3), $h_{i}^{\left(l\right)}$ denotes the node hidden state at layer l, $h_{ij}^{e}$ denotes the fixed output of the edge encoder, and N(j) denotes the neighborhood of node *j*. The functions $\phi_{e}^{\left(l\right)}$ and $\phi_{v}^{\left(l\right)}$ represent the message construction and node update networks, respectively, and || denotes feature concatenation. Eight residual message-passing layers are used to capture coupling between the gauge and transition regions while limiting the excessive smoothing and loss of high-frequency field structure that can occur in deeper graph networks. The effectiveness of deep graph architectures for solid-mechanics field prediction has been demonstrated for a range of metallic structures [5,7].

### 3.4. Field Decoder and Independent Structured Force Branch

The field decoder maps the final nodal hidden features to the von Mises stress field and the three displacement components. A graph-level guide-force head is used only during pretraining to provide a global loading constraint and is not used as the final force-curve output.

The independent force branch uses only normalized displacement and material conditions. It represents the complete force–displacement response through a two-timescale saturating baseline, a bounded residual term, and a terminal load-drop gate, as defined in Equation (4). Because this branch does not read features from the graph backbone, subsequent force-curve optimization does not alter the spatial-field parameters.

(4) $$\hat{F}\left(\tilde{\delta }\right)=\max\left\{F_{\mathrm{base}}\left(\tilde{\delta }\right)+F_{\mathrm{res}}\left(\tilde{\delta }\right),0\right\}s\left(\tilde{\delta }\right)$$

In Equation (4), *F*_base_ denotes the two-timescale saturating baseline, *F*_res_ is the bounded residual term, $s\left(\tilde{\delta }\right)$ and is the terminal load-drop gate. This gate is used only to parameterize the load-drop shape within the prescribed loading path and training distribution; it is not interpreted as a predictor of crack-initiation time under unknown geometries or arbitrary defect conditions. The mechanical response of additively manufactured AlSi10Mg is known to depend strongly on build orientation, with appreciable differences in load-drop and fracture behavior among orientations [21]. The two saturation amplitudes and rates govern the fast and slow load build-up, the linear contribution represents a hardening-like slope, and the critical-displacement and sharpness parameters define the terminal sigmoid gate.

### 3.5. Pretraining Loss and Two-Stage Optimization of the Force Branch

In Stage I, the field backbone is trained with the multitask objective in Equation (5), which jointly constrains nodal stress, displacement, frame-level reaction force, graph-level guide force, and spatial regularization. Stress prediction is treated as the primary task, while the auxiliary terms improve displacement consistency, global load consistency, spatial smoothness, and physical consistency near the zero-load state.

(5) $$L_{pre}=2.00L_{\sigma }+0.10L_{u}+0.20L_{F,frame}+0.50L_{F,guide}+0.10L_{\Delta u}+0.05L_{\Delta \sigma }+5.00L_{0}$$

Here, *L_σ_*, *L_u_*, and *L_F,frame_* are the mean-squared-error terms for nodal von Mises stress, three-component displacement, and frame-level reaction force, respectively. *L_F,guide_* supervises the graph-level auxiliary guide-force head during Stage I; it supplies a global load constraint to the field backbone but is not used as the final force–displacement output. *L*_Δ_*_u_* and *L*_Δ_*_σ_* regularize spatial variations in the displacement and stress fields to suppress nonphysical local oscillations, while *L*_0_ enforces consistency near the initial zero-load state. After convergence of the field backbone, training proceeds to Stage II, in which the GNN and field decoder are frozen and only the independent structured force–displacement branch is optimized. This branch takes normalized displacement and material conditions as its inputs and does not use graph-level features obtained by pooling GNN node representations. Its training objective is constructed for the complete force–displacement curve and jointly considers pointwise error, local slope and curvature, peak load, and terminal load-drop characteristics to preserve both the magnitude and shape of the predicted response. The structured gate describes only the terminal load decrease along the prescribed loading path and is not used to predict crack initiation under unknown conditions. Related multitask neural network optimization strategies have been applied to low-cycle fatigue life prediction, where genetic algorithm optimization of network weights improved predictive accuracy [22]. Post-processing also has a strong influence on micropore evolution and mechanical properties in additively manufactured AlSi10Mg, and process-induced variability should therefore be considered when interpreting model performance [23].

## 4. Training Strategy and DIC Transfer

### 4.1. Simulation-Domain Pretraining and Model Selection

The graph-field backbone is trained using the AdamW optimizer with an initial learning rate of 1 × 10^−4^, together with learning rate decay, early stopping, and automatic mixed precision. Model selection is based on nodal stress error and performance in high-stress regions on the validation set. After the best field model is obtained, the graph-field backbone is frozen and the independent force branch is optimized in Stage II. The learning rate and early stopping of the force branch are controlled by the complete-curve error on the validation set, and the field backbone parameters are verified to remain unchanged after force-branch training. The GNN and the MLP baseline were implemented in PyTorch (version 1.12.1) with CUDA 11.3.

For a simple non-graph comparison, a node-wise conditional multilayer perceptron (MLP) was trained on the same complete-FE-case split and stress target. The baseline uses the same 32-dimensional static nodal features and 14-dimensional displacement/material condition vector, but no graph edges, neighborhood message passing, graph pooling, or FiLM modulation. The four-hidden-layer, 128-unit MLP contains 56,705 trainable parameters and is selected by validation RMSE before evaluation on the same nine held-out FE cases.

### 4.2. Experimental DIC Data and Screenshot Sequence Organization

**Figure 3:**
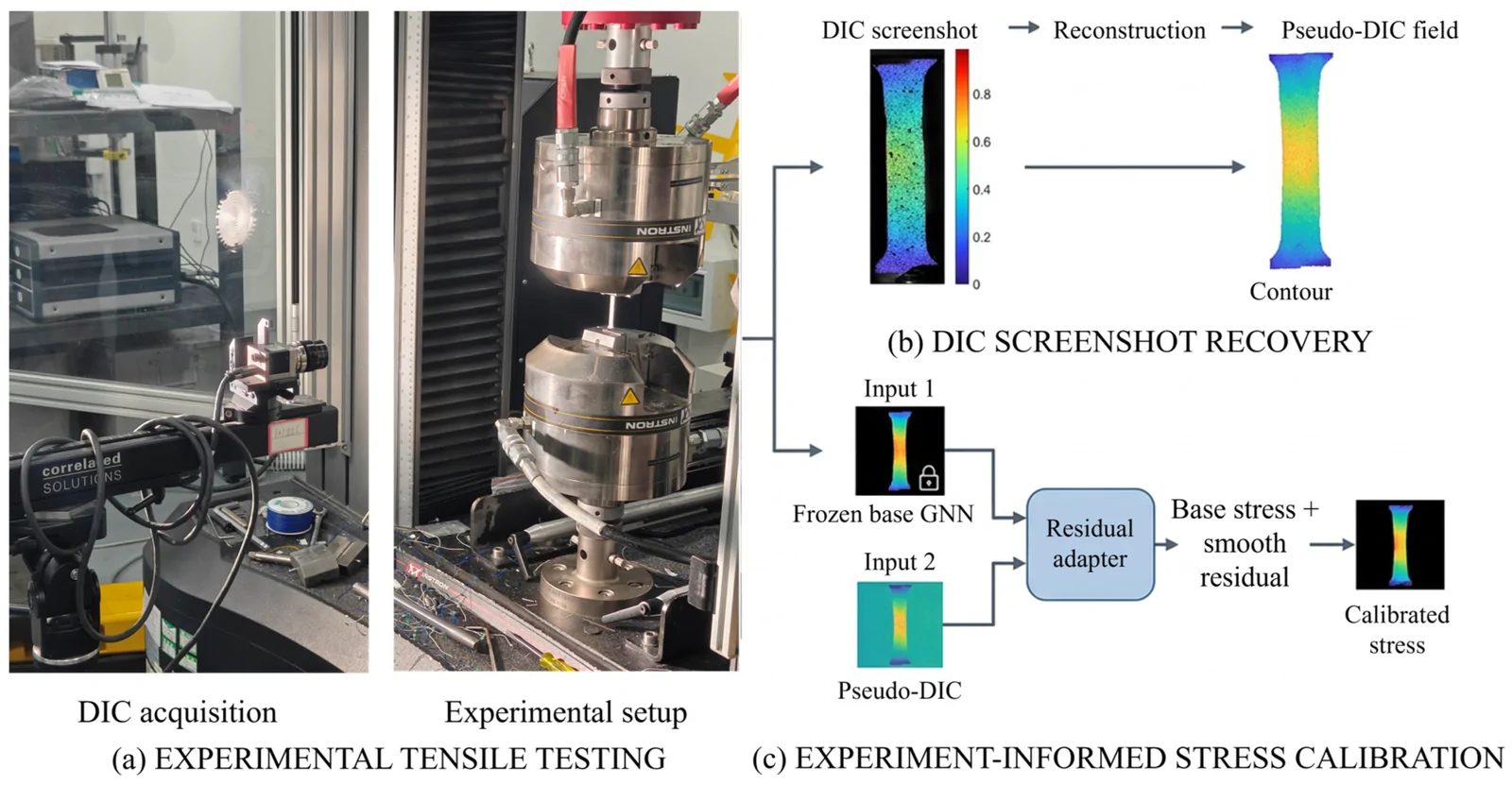
Experimental tensile testing, DIC screenshot recovery, and DIC-informed spatial regularization: (**a**) tensile testing and DIC acquisition; (**b**) DIC screenshot recovery and pseudo-field construction; and (**c**) weakly supervised spatial regularization with the pretrained GNN frozen.

The experiments comprised eight AlSi10Mg tensile specimens. The tensile testing setup, specimen mounting, and DIC acquisition procedure are illustrated in Figure 3a. During loading, the DIC system recorded the surface deformation response and exported longitudinal engineering-strain contour screenshots sequentially, whereas the original numerical matrices were not retained. Because the color scale varies from frame to frame, the vertical color bar is detected independently for each screenshot and a 256-level color look-up table is reconstructed. Relative field values are recovered through CIELAB color matching, while textual annotations, measurement lines, and local artifacts are removed [8]. After reconstruction of the complete specimen contour and completion of missing regions, each frame is converted into a 384 × 128 normalized pseudo-DIC field bounded to [0, 1], together with a valid mask and confidence map. Because each frame has an independent display range, the recovered field represents only the relative spatial pattern within that frame and is not interpreted as an absolute strain or stress label that can be compared quantitatively across frames. Accordingly, pseudo-DIC fields provide only relative spatial cues and cannot be used for quantitative strain or stress validation. The eight specimens contain 25,645 source screenshots; fixed-interval sampling and quality control retain 2509 pseudo-DIC samples. The weighted mean image quality score is 0.959, and the reliable pixel ratio is 0.809. Deep-learning-based full-field surface-strain analysis provides a promising route for online damage monitoring [8,9,24,25].

After color scale recovery, image cleaning, and specimen contour reconstruction, each screenshot was converted into a normalized pseudo-DIC field while retaining the valid mask and confidence information, as illustrated in Figure 3b.

Color recovery uses the direction of the CIELAB chroma vector rather than absolute RGB distance. Direction confidence is combined with chroma confidence, and a pixel is treated as directly observed only when it lies inside the specimen mask, has chroma ≥ 7, brightness V ≥ 18, and confidence ≥ 0.18; a 3 × 3 morphological opening removes isolated mismatches. Missing in-specimen regions are completed from the nearest valid pixels, followed by a 3 × 3 median filter and mild anisotropic Gaussian smoothing. The reported reliable-pixel ratio is the fraction of specimen-mask pixels that satisfy the valid-pixel criteria. The frame quality score combines valid fraction, mean confidence, specimen-shape consistency, and boundary completeness with weights 0.38, 0.27, 0.20, and 0.15, respectively.

The archived screenshot sequences were not synchronized frame-by-frame to machine timestamps. Each experimental sequence was therefore associated with displacement through monotonic sequence progress within the specimen loading range; this is a sequence-based association rather than exact timestamp synchronization. The screenshot-reconstruction pipeline was additionally tested on 72 synthetic screenshots generated from known normalized spatial fields with color bars and controlled degradations, including annotation/occlusion, compression, brightness/color perturbation, and combined degradation. The same recovery, masking, confidence weighting, missing-region completion, and smoothing operations were applied without access to the underlying reference field.

### 4.3. Finite-Element Surface Mapping and Low-Frequency Stress Residual Adapter

The transfer stage uses the same shared FE topology as simulation-domain pretraining, from which the front visible surface of the specimen is extracted. For each experimental frame, the base GNN is queried at the sequence-associated displacement to obtain nodal von Mises stress. The DIC information is mapped to a regular grid only for loss evaluation; the final calibrated values are written back to the FE surface nodes and visualized on the original triangular mesh. The front FE surface coordinates are normalized to the specimen bounding box, linearly rasterized to the same 384 × 128 grid used by the pseudo-DIC fields for loss evaluation, and the learned residual is bilinearly sampled back to the original FE surface nodes for visualization.

The residual adapter takes the base stress, pseudo-DIC field, specimen mask, and longitudinal coordinate as inputs. Its low-frequency residual construction and stress-regularization formulation are given in Equation (6).

(6) $$\begin{array}{l} R=P_{17\times 9}\left\{16\tanh\left[A_{\psi }\left(I\right)\right]\right\}\\ \Delta \sigma =M⊙\left[R-\mu_{M}\left(R\right)\right]\\ \sigma_{\mathrm{cal}}=\mathrm{clip}\left(\sigma_{\mathrm{base}}+\Delta \sigma ,0,396\;\mathrm{MPa}\right) \end{array}$$

In Equation (6), $A_{\psi }$ (·) denotes the residual-adapter mapping, P_17×9_ represents 17 × 9 average pooling, M is the valid specimen mask, and *μ_M_*(·) denotes the mean over valid pixels. The candidate residual is first generated through a tanh mapping with a nominal scale of 16 MPa, followed by 17 × 9 average pooling and masked mean removal. Therefore, 16 MPa is a nominal scale used for residual generation rather than a strict pointwise bound on the final correction after mean removal. The calibrated stress is finally clipped to 0–396 MPa. This design focuses transfer on spatial redistribution while limiting excessive modification of the overall stress level by experimental image noise. Pore defects in laser-deposition-fabricated AlSi10Mg can strongly modify local stress concentrations, and crystal-plasticity FE analysis has shown that peak stresses near pores may reach several times the nominal stress [26]. The 16 MPa scale, tanh saturation, and 17 × 9 pooling are regularization-oriented modeling choices rather than AlSi10Mg material constants or quantities inferred from a DIC noise spectrum. They bound extrapolative residuals and suppress high-spatial-frequency screenshot artifacts so that the experimental branch perturbs rather than replaces the simulation prior.

Because the pseudo-DIC field and stress have different physical dimensions, training does not compare their absolute values. Instead, weak supervision is established through confidence-weighted standardized spatial patterns. The objective in Equation (7) combines experimental spatial-pattern constraints, preservation of the base prediction, and physical regularization. The shape and gradient losses constrain the relative spatial distribution and local direction of variation; the identity preservation term limits the residual amplitude; and the total variation, zero mean, and symmetry terms suppress high-frequency noise, global stress drift, and nonphysical transverse bias, respectively.

The screenshot-derived longitudinal strain pattern and the von Mises stress field are not physically equivalent observables. Under the fixed monotonic tensile configuration, their relative localization morphology can provide a weak spatial cue, but the strain–stress relationship becomes non-unique after yielding, necking, and damage localization. The pseudo-DIC field is therefore used only to constrain relative spatial ordering and gradient morphology; it is never treated as an experimental stress label or as quantitative strain ground truth.

Operationally, the shape term compares confidence-weighted standardized (z-scored) spatial fields using a Smooth-L1 penalty, the gradient term compares horizontal and vertical finite differences, the identity term penalizes the normalized residual amplitude, and the total-variation term penalizes first spatial differences. The zero-mean and transverse-symmetry terms suppress masked mean drift and nonphysical transverse bias, respectively; these definitions correspond directly to the components of Equation (7).

(7) $$L_{\mathrm{weak}}=0.14L_{\mathrm{shape}}+0.04L_{\mathrm{grad}}+1.00L_{\mathrm{id}}+0.14L_{\mathrm{TV}}+0.30L_{\mathrm{mean}}+0.08L_{\mathrm{sym}}$$

Accordingly, the adapter is not intended to reconstruct a stress field directly from DIC screenshots. Its role is to perform bounded, smooth, low-frequency spatial regularization while preserving the simulation-derived mechanics prior. Both the base GNN and the structured force–displacement branch remain frozen throughout transfer. The resulting field should therefore be interpreted as simulation-derived stress with experiment-informed spatial regularization, not as experimentally measured or independently validated stress.

### 4.4. Ablation Study Design

Controlled ablation studies are conducted for both the simulation-domain GNN and the DIC residual adapter to quantify the contribution of individual modules. All variants use the same evaluation metrics and test protocols, and only the target component is changed. The ablations are intended to identify the relative role of each module rather than to broaden the applicability of the conclusions beyond the current material parameter range, geometry, or defect location. Four simulation-domain variants are considered: removing FiLM conditioning from the message-passing layers; reducing the processor depth from eight to four layers; removing the training-only guide-force regularization; and omitting Stage II force-branch fine-tuning while retaining the Stage I configuration. The complete model serves as the baseline. Four DIC adapter variants are also evaluated: removing both shape and gradient supervision; removing identity preservation; removing gradient consistency while retaining spatial shape matching; and removing the 17 × 9 low-pass average pooling. DIC ablations are repeated with multiple random seeds, and statistics are reported on the independent L-7 and L-8 test specimens. Studies of AlSi10MnMg produced with recycled feedstock have shown that the relationship between microstructure and mechanical properties is highly sensitive to processing parameters, underscoring the value of systematic ablation when assessing model robustness [27]. The no-FiLM variant removes the principal layer-wise condition-injection mechanism in this architecture and is not intended as an exhaustive comparison against all possible conditioning strategies.

## 5. Results and Discussion

### 5.1. Simulation-Domain Full-Field Response Prediction

Simulation-domain performance is evaluated on an independent test set split by complete FE cases (Table 2). The test set contains nine FE cases that were not used for training or model selection, corresponding to 252 loading frames. Because all loading states from a given case remain within the same subset, leakage caused by placing adjacent loading frames in different datasets is avoided. Given the material parameters and loading displacement, a single forward evaluation simultaneously returns nodal von Mises stress, three-component displacement, and the corresponding global loading force. Across all test nodes, the von Mises stress prediction achieves R^2^ = 0.9984, with an RMSE of 4.60 MPa and an MAE of 1.80 MPa. Frame-wise evaluation yields a mean R^2^ of 0.9822 and a relative L2 error of 3.95%, indicating that the model achieves high global nodal accuracy while consistently reconstructing the spatial response across different loading stages. The RMSE of the nodal displacement prediction is 0.0309 mm. Prediction errors increase moderately as loading progresses into plastic localization and damage evolution. The stress RMSEs in hotspot regions, late loading stages, and damage-adjacent regions are 7.32, 9.91, and 11.80 MPa, respectively. This increase is associated mainly with stronger stress gradients near the centrally weakened region, rapid evolution of the damage variable, and field discontinuities introduced by element deletion. Despite the larger local errors, the model still identifies the locations of high-stress regions and their principal evolution trends. The high accuracy of graph neural networks for reconstructing full-field mechanical responses has also been demonstrated for a range of metallic structures [5,7].

**Figure 4:**
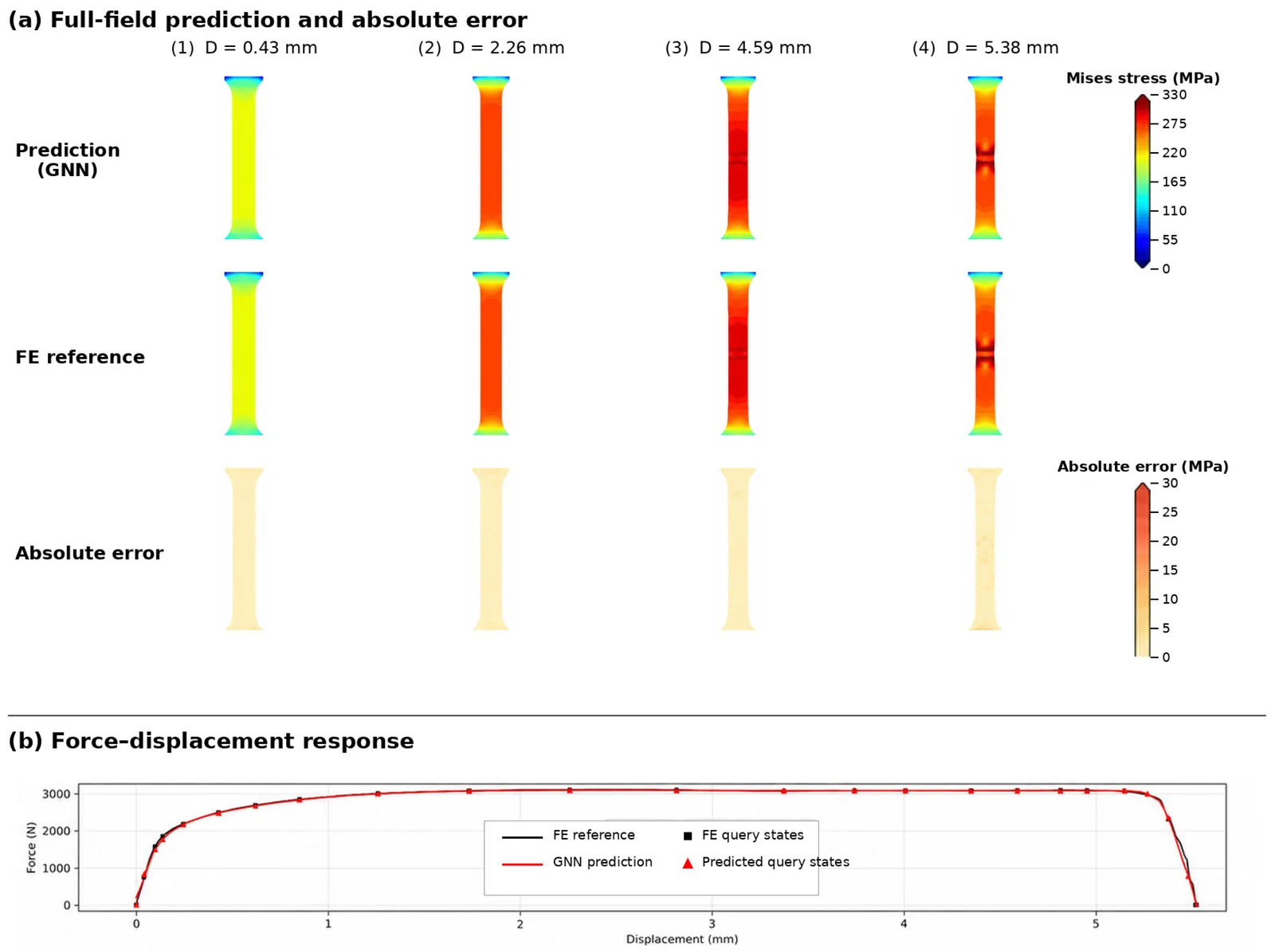
Comparison of von Mises stress predictions and force–displacement responses at representative loading stages.

Figure 4 presents predictions for a representative test case at several loading stages. During the elastic and stable plastic regimes, the GNN prediction agrees closely with the FE reference in both stress magnitude and spatial distribution. As the response approaches peak load, a pronounced stress concentration develops near the specimen center, and the model accurately captures the localization position and stress gradient. During the rapid terminal loss of load-carrying capacity, the largest errors are concentrated within and around the damage band. The absolute-error fields remain low overall, indicating that graph message passing effectively learns local interactions on the FE mesh and their evolution with loading conditions. At the stored FE loading frames, the force predictions yield R^2^ = 0.9873, RMSE = 139.08 N, and MAE = 45.59 N. For the dense force–displacement curve obtained by continuous queries of the structured force branch, R^2^ increases to 0.9957 and RMSE decreases to 59.70 N. The predicted curve reproduces the main trends in initial stiffness, plastic hardening, peak load, and the near-terminal load-drop stage. The stored-frame and dense-curve metrics correspond to different sampling protocols: the former quantify agreement at FE output states, whereas the latter more directly assess continuous reconstruction of the complete response by the structured force branch. After model loading, the mean inference time for simultaneous prediction of the nodal stress field, displacement field, and loading force at a single displacement state is 29.88 ± 0.92 ms. A complete 201-point force–displacement curve is generated by the independent structured force branch in only 3.07 ± 0.07 ms on average. These timings exclude initial model loading and data preprocessing. Because Abaqus wall-clock times were not retained under the same hardware and timing protocol, no FE-to-GNN speedup factor is reported to avoid an unreliable comparison based on inconsistent timing scopes.

Additional FE and experimental validation were performed to further assess the numerical reliability of the proposed framework. The representative explicit-dynamics check gave a maximum ALLKE/ALLIE ratio of 0.352% over the principal load-bearing interval (0.14–5.50 mm). In the 0.50/0.40/0.35 mm mesh study, peak forces differed by less than 0.12%; before terminal deletion, the 0.50 mm production mesh differed from the 0.35 mm reference by 0.24% normalized force-curve RMSE up to 5.0 mm, and the maximum absolute differences in global mean and P95 von Mises stress at the evaluated pre-terminal displacements were 0.94% and 0.50%, respectively. The exact terminal deletion time was non-monotonic with mesh size, so strict convergence of fracture/deletion timing is not claimed.

On the same held-out FE cases, the non-graph node-wise conditional MLP achieved R^2^ = 0.9932 and RMSE = 9.56 MPa, compared with R^2^ = 0.9984 and RMSE = 4.60 MPa for the GNN. The MLP hotspot, late-stage, and damage-zone RMSEs were 15.77, 18.52, and 42.78 MPa, versus 7.32, 9.91, and 11.80 MPa for the GNN. Thus, the fixed-topology problem is comparatively structured, while graph message passing provides a measurable additional benefit, especially near damage localization.

**Figure 5:**
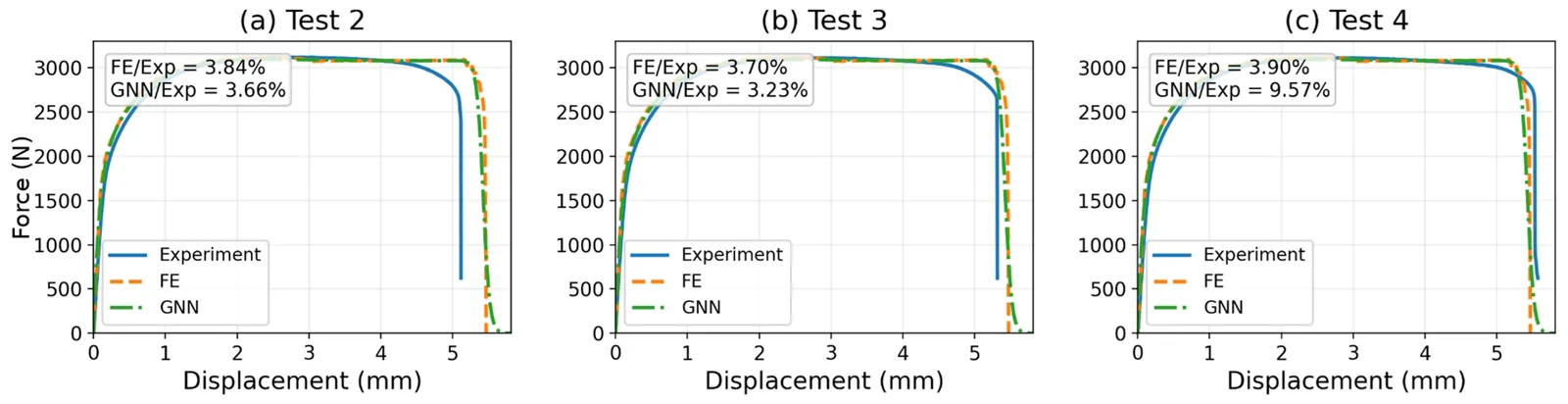
Machine experiment, FE reference, and trained GNN force-branch comparisons.

Machine force–displacement data from five tensile tests were compared with both the corresponding FE responses and the trained structured force branch. The force branch was queried directly at the nominal material condition used for this experimental-series comparison; no specimen-specific inverse identification was performed. For the FE curves, post-deletion Explicit reaction force oscillations were excluded. The GNN curve is the raw force-branch output without display smoothing or manual terminal-drop correction. For compact visualization, Figure 5 retains Tests 2–4, which were selected previously because they exhibited the closest FE-experiment terminal displacement agreement while retaining FE experiment whole-curve NRMSE below 4%. Across all five cases, the FE experiment curve NRMSE averaged 4.40% (maximum 6.77%), while the GNN experiment curve NRMSE averaged 5.31% (maximum 9.57%). The peak-force error averaged 0.22% for FE and 0.55% for the GNN force branch (maximum 0.60% and 0.81%, respectively). The FE fracture displacement error averaged 7.80% (maximum 14.20%). The larger GNN discrepancy in Test 4 is concentrated near terminal failure, where the learned structured gate produces a smoother load decline than the abrupt experimental termination. These comparisons provide independent experimental support for the global response represented by both the FE reference and the learned force branch, but they do not directly validate the local FE/GNN stress field or specimen-specific fracture timing.

The same nominal-condition raw GNN curve is shown in each panel; no display smoothing or manual terminal-drop correction is applied. The reported NRMSEs are evaluated over each experiment’s recorded pre-fracture interval. FE reaction force oscillations after terminal element deletion are excluded.

For completeness, the FE experiment curve NRMSEs for Tests 1–5 were 3.80%, 3.84%, 3.70%, 3.90%, and 6.77%, respectively; the corresponding GNN experiment values were 3.45%, 3.66%, 3.23%, 9.57%, and 6.64%. The two cases not displayed in Figure 5 were Test 1 (FE peak-force error 0.17%, FE fracture displacement error 14.20%) and Test 5 (0.60% and 13.14%, respectively). The direct GNN comparison therefore confirms good global force response agreement overall while also exposing the expected limitation in reproducing specimen-specific terminal failure timing with a single nominal-condition force curve.

**Figure 6:**
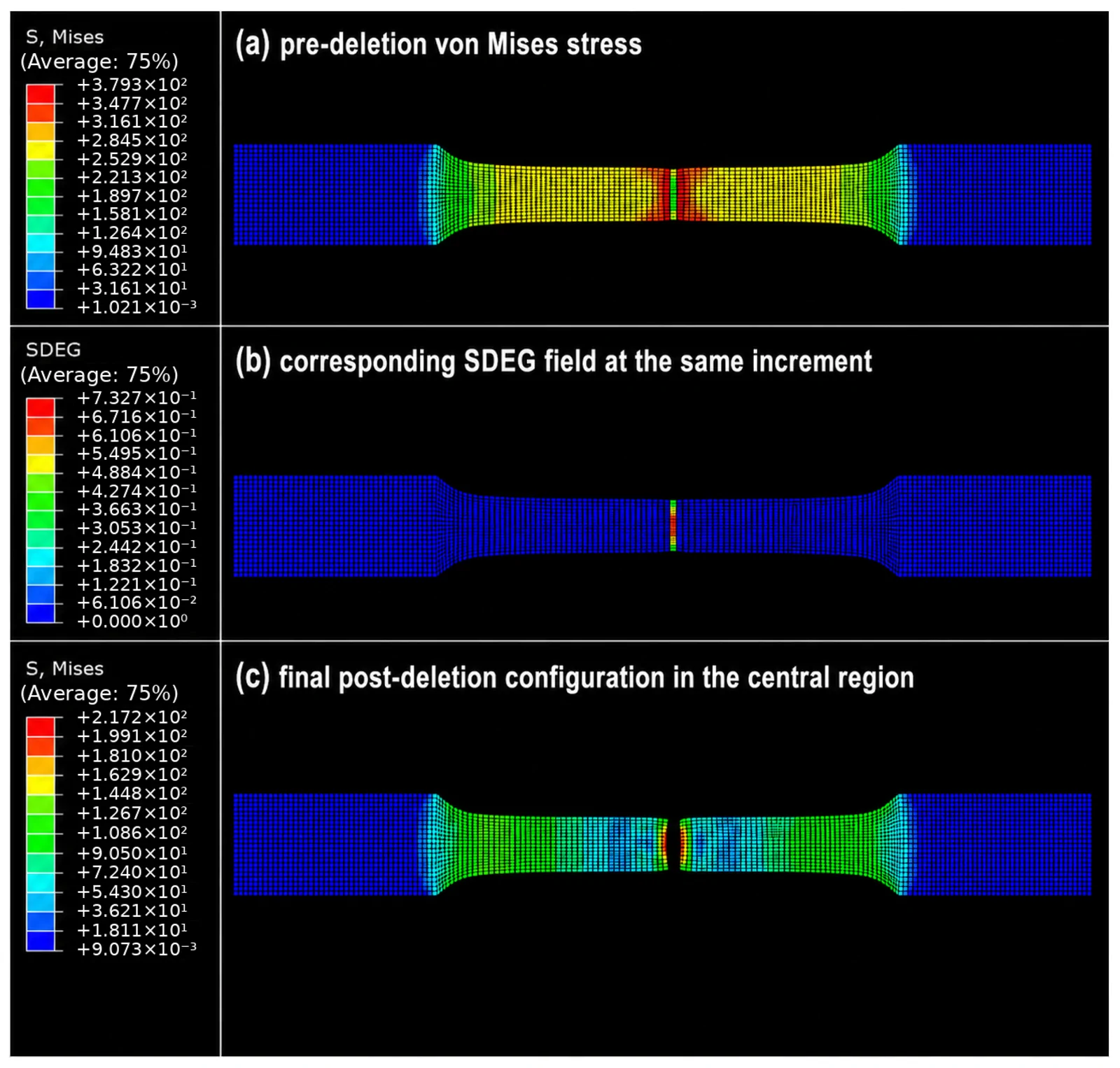
Representative FE failure evolution.

The implemented failure mechanism is shown in Figure 6. Immediately before terminal deletion, the von Mises stress localizes in the prescribed central weakened band and the corresponding SDEG field reaches approximately 0.885 at the same increment. The final configuration then shows localized separation after element removal. This visualization documents the implemented damage/deletion process; because the weakened band is prescribed, it should not be interpreted as prediction of an arbitrary crack-initiation location.

### 5.2. DIC Screenshot Reconstruction and Weakly Supervised Spatial Regularization

The eight experimental specimens contain 25,645 DIC source images with frame-specific color bars. After color bar parsing, CIELAB color matching, artifact removal, full-contour reconstruction, and fixed-interval screening, 2509 valid pseudo-DIC field samples are retained. The mean image quality score is 0.959 and the reliable pixel ratio is 0.809. Transfer uses a specimen-level split: L-1 to L-5 are used for training, L-6 for model selection, and L-7 and L-8 as independent test specimens. The base GNN and the structured force–displacement branch remain frozen throughout transfer, and only the low-frequency stress residual adapter is trained. High-cycle and very-high-cycle fatigue responses of additively manufactured AlSi10Mg are known to be sensitive to microstructure and residual stress, and near-surface behavior can vary markedly with post-processing condition [28,29,30].

The synthetic screenshot benchmark succeeded in all 72/72 cases. For recovery of the normalized relative spatial field, the mean RMSE was 0.0659, the mean Pearson and Spearman correlations were 0.9698 and 0.9540, the mean gradient MAE was 0.00613, and the mean mask IoU was 0.944. Under the combined-degradation condition, Spearman correlation remained 0.939 and mask IoU 0.903. These results support recovery of relative spatial morphology under the tested screenshot degradations; they do not validate absolute DIC strain or stress.

**Figure 7:**
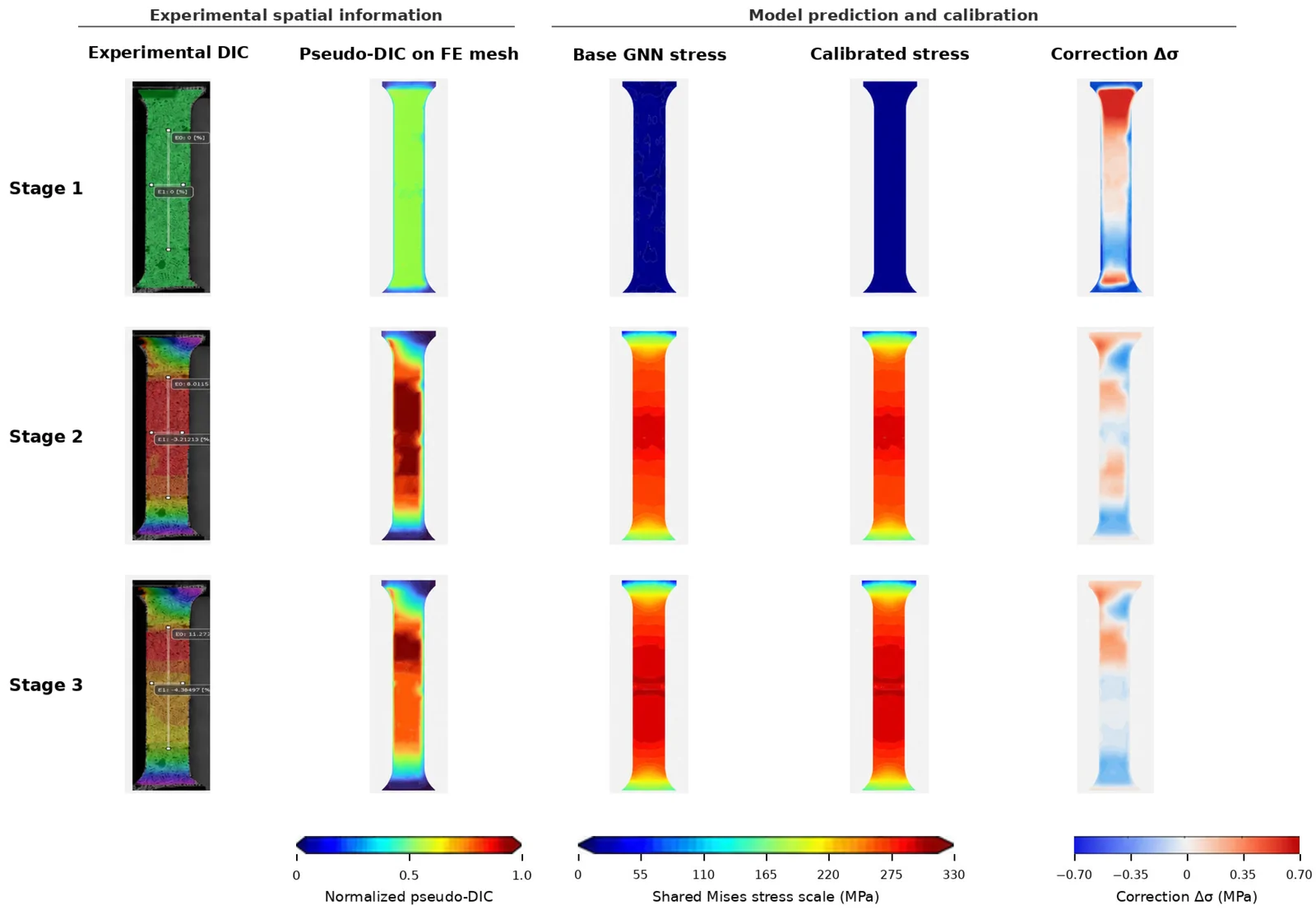
Weakly supervised DIC-informed spatial regularization results.

On the independent L-7 and L-8 test sets, the DIC spatial shape loss decreases from 0.2405 to 0.2328, corresponding to a 3.21% improvement, while the spatial gradient loss improves by 1.92%. The mean absolute stress correction is 0.232 MPa, the 95th percentile is 0.636 MPa, and the maximum correction observed across all exported test fields is approximately 6.20 MPa, substantially below the nominal 16 MPa residual-generation scale. Representative loading stages (Figure 7) show that the pseudo-DIC field retains the principal spatial trends visible in the screenshots, whereas the base GNN provides the stress magnitude and overall evolution consistent with the FE prior. After residual adaptation, the dominant spatial pattern of the regularized stress field remains close to the base prediction, while the correction appears as a continuous, low-amplitude, spatially structured adjustment. This behavior is consistent with the intended strategy of spatial regularization rather than reconstruction. The small visual difference between the base and calibrated fields is consistent with the mean correction magnitude of 0.232 MPa; it does not imply that the experimental information is ineffective but rather reflects the strong preservation constraint placed on the simulation prior. Because the base GNN is already highly accurate in the simulation domain, the role of transfer is to reduce discrepancies in spatial distribution using experimental image information rather than to obtain an apparent loss reduction by increasing the correction magnitude. Defect volume and location strongly affect the very-high-cycle fatigue life of laser-powder-bed-fused AlSi10Mg, highlighting the importance of reliable local stress characterization [30]. Relative to a representative stress scale of approximately 330 MPa, the mean, 95th-percentile, and maximum corrections correspond to about 0.07%, 0.19%, and 1.9%, respectively. The deliberately small amplitude is consistent with spatial regularization rather than quantitative stress re-estimation; accordingly, the reported 3.21% improvement is a DIC-consistency/spatial shape metric, not a 3.21% improvement in physical stress accuracy.

### 5.3. Module Ablation Studies

#### 5.3.1. Simulation-Domain GNN Ablation

**Figure 8:**
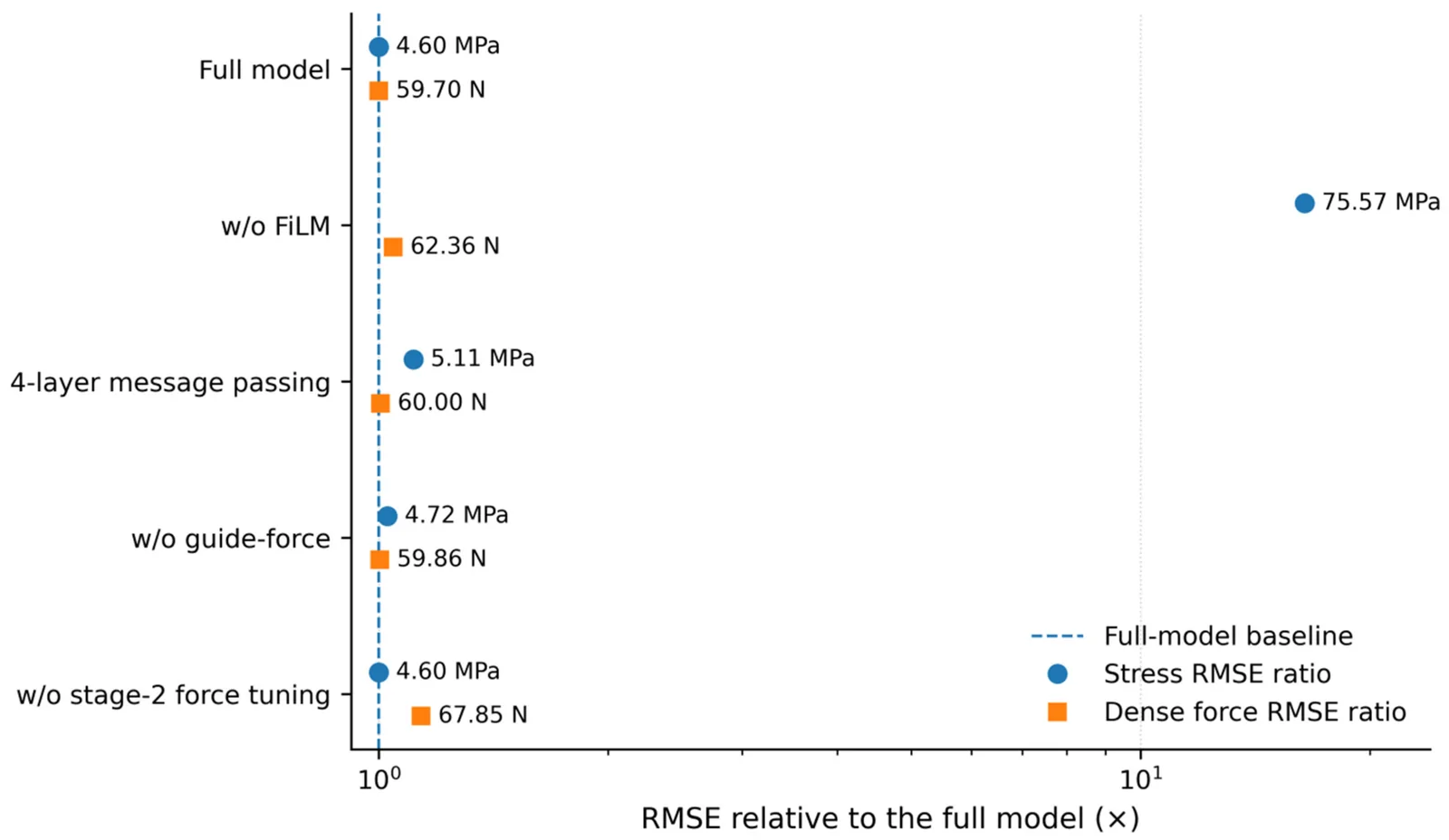
Ablation results for key modules of the simulation-domain GNN.

Figure 8 compares the complete model with four simulation-domain variants. Removing FiLM reduces the stress R^2^ from 0.9984 to 0.5783 and increases the RMSE from 4.60 to 75.57 MPa, demonstrating that layer-wise condition modulation is essential for representing loading-stage-dependent full-field responses. Reducing the message-passing depth from eight to four layers yields a stress RMSE of 5.11 MPa, representing a modest degradation and indicating that deeper propagation helps integrate mechanical coupling across local mesh neighborhoods and between the gauge and transition regions. Removing guide-force regularization gives a stress RMSE of 4.72 MPa, showing that this training-only global load constraint provides auxiliary benefit but is not the primary source of field accuracy. When Stage II force-branch fine-tuning is omitted, the stress metrics remain essentially unchanged, whereas the dense force-curve RMSE increases from 59.70 to 67.85 N. This result directly supports the field-force decoupling strategy: Stage II optimization improves reconstruction of the complete force–displacement curve without rewriting the learned spatial fields. Strength assessment of additively manufactured metals in the very-high-cycle fatigue regime has become an active research topic, for which surrogate accuracy and robustness directly affect the reliability of fatigue life prediction [31,32]. The no-FiLM variant removes the principal layer-wise loading/material condition injection mechanism in this architecture; its deterioration demonstrates the importance of explicit conditioning here but does not establish FiLM as universally superior to other conditioning strategies.

#### 5.3.2. Ablation of the Weakly Supervised DIC Adapter

As summarized in Table 3, the DIC adapter ablations are repeated with multiple random seeds and evaluated on the independent L-7 and L-8 test specimens. Under this controlled ablation protocol, the complete adapter yields a 3.35% ± 0.07% improvement in spatial shape loss and a mean absolute stress correction of 0.224 MPa. The 3.35% value is the multi-seed ablation mean, whereas the 3.21% reported in Section 5.2 corresponds to the final model in the main experiment; the two values therefore use different statistical protocols but are of comparable magnitude. Removing DIC shape and gradient supervision reduces the improvement to −0.09% and gives zero correction amplitude, confirming that the experimental spatial pattern is the direct source of spatial-consistency gain. Removing identity preservation increases the apparent shape-loss improvement to 58.14%, but the mean absolute stress correction simultaneously increases to 9.021 MPa and the normalized residual TV rises to 0.019839. This variant cannot be interpreted as a better model: when spatial consistency is optimized without preservation of the base prediction, the adapter can obtain a large apparent reduction in shape loss by substantially rewriting the base stress field. Identity preservation is therefore critical for preventing over-correction. Removing gradient consistency gives a 3.26% improvement, close to that of the complete adapter, indicating that its main contribution is an auxiliary constraint on the direction of local spatial variation. Removing the 17 × 9 low-pass pooling slightly increases the shape-loss improvement to 3.59%, but the residual TV rises from 0.000667 to 0.000810. Thus, a marginally better spatial match does not necessarily correspond to a more physically credible spatial regularization; the low-frequency constraint trades a very small amount of shape-loss performance for a smoother and more interpretable residual field. Considering experimental consistency, correction magnitude, and spatial smoothness together, the complete adapter provides the most balanced behavior. Direct aging can substantially modify the microstructure, mechanical properties, and residual stress of selective-laser-melted AlSi10Mg, emphasizing the importance of controlled material condition when interpreting mechanical response [33]. The no-identity and no-low-pass variants therefore also serve as sensitivity evidence that the adapter design intentionally favors bounded, smooth corrections rather than the largest possible surrogate-loss reduction.

### 5.4. Effect of the Number of Training Specimens and Overall Discussion

**Figure 9:**
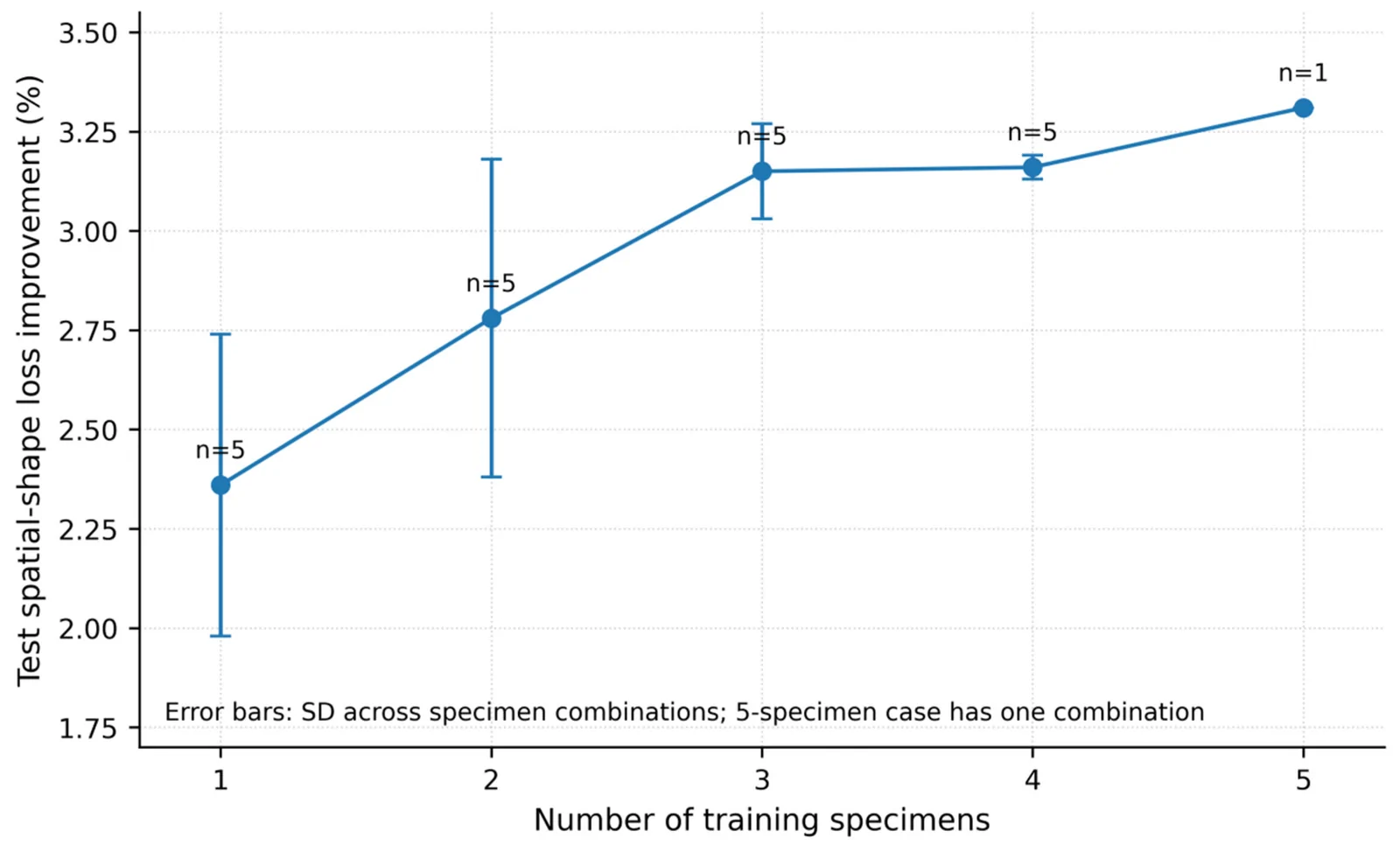
Effect of the number of training specimens on the improvement in spatial shape loss for the independent test set.

Figure 9 shows the effect of the number of training specimens on independent-test performance. Increasing the number of training specimens from one to five reduces the spatial shape loss after regularization from 0.2348 to 0.2325 and increases the improvement from 2.36% to 3.31%. With three training specimens, the improvement already reaches 3.15%; adding further specimens continues to improve the mean performance, but with diminishing marginal gains. The reduction in variability between specimen combinations is more pronounced than the improvement in the mean. The standard deviation of the calibrated shape loss decreases from 0.000919 for single-specimen training to 0.000082 for four-specimen training. This result indicates that additional physically independent specimens reduce sensitivity to the speckle quality, imaging conditions, and local correlation failures of any single specimen; improved transfer stability is therefore a major benefit of multi-specimen training. Across the tested training-set sizes, the mean absolute stress correction remains within 0.200–0.236 MPa and does not increase monotonically with the number of specimens. The performance gain is thus associated primarily with stabilization of the residual spatial pattern rather than with larger corrections. Correction magnitude itself is not a monotonic performance metric, and a larger correction does not necessarily imply more reliable spatial regularization. Reviews of high-cycle and very-high-cycle fatigue in laser-powder-bed-fused Al-Si alloys likewise emphasize the importance of multi-specimen statistics for mitigating process-induced scatter and establishing reliable mechanical property assessment [34].

Taken together, the main experiments, module ablations, and training specimen analysis reveal two coupled levels in the proposed framework. The simulation-domain GNN rapidly reconstructs stress, displacement, and the global load response, whereas the DIC residual adapter introduces experimental spatial information after the base model has been frozen. FiLM conditioning and sufficient message-passing depth support loading-stage-dependent full-field representation, while Stage II force-branch optimization improves the complete force curve. DIC shape and gradient supervision provide the experimental driving signal, whereas identity preservation and low-frequency constraints jointly limit correction magnitude and high-frequency noise. Three limitations should be emphasized. First, the current FE parameter variation is dominated by elastic modulus and does not yet cover multi-source uncertainty in yield strength, hardening, damage parameters, or geometric defects. Second, the experimental input consists of frame-wise screenshots; the DIC fields reconstructed from the color bars provide only relative spatial patterns and cannot replace original DIC displacement or strain matrices with absolute physical units. Third, the current specimens have a fixed geometry and a prescribed centrally weakened region, and generalization across geometries, defect locations, and arbitrary crack paths remains to be established. Within these limitations, the results support a conservative simulation-experiment fusion strategy in which the simulation-domain mechanics prior is retained, the base predictor is frozen, and only a constrained low-frequency residual is learned, rather than retraining the full-field model directly from screenshots. Post-heat treatment can strongly modify the microstructure and mechanical properties of selective-laser-melted AlSi10Mg, and future validation should therefore consider a broader material-parameter space [35]. The added machine force–displacement comparison supports both the global FE response and the trained force branch, with mean pre-fracture curve NRMSEs of 4.40% and 5.31%, respectively. Because the force branch was queried at a common nominal material condition rather than specimen-specific identified parameters, this comparison is a global-response check rather than a specimen-specific inverse calibration. It does not directly validate the local FE/GNN stress field. In addition, the exact terminal element deletion time is mesh-sensitive, so no claim of mesh-converged fracture timing is made.

## 6. Conclusions

This work addresses the high computational cost of nonlinear full-field FE analysis for AlSi10Mg tensile specimens and the absence of original numerical DIC fields by developing a full-field stress prediction framework that combines a simulation-domain GNN, pseudo-DIC field reconstruction from screenshots, and weakly supervised low-frequency DIC-informed spatial regularization. The main conclusions are as follows.

1. The conditioned GNN simultaneously predicts nodal von Mises stress, three-component displacement, and the global force–displacement response. Across 252 loading frames from nine independent test cases, the stress prediction achieves R^2^ = 0.9984, RMSE = 4.60 MPa, and MAE = 1.80 MPa. The dense force curve achieves R^2^ = 0.9957 and RMSE = 59.70 N. The joint query time for the full field and loading force at a single displacement state is 29.88 ± 0.92 ms, while a complete 201-point force curve is generated in 3.07 ± 0.07 ms. Five machine-recorded tensile curves independently support the global response: the mean pre-fracture curve NRMSE is 4.40% for FE versus experiment and 5.31% for the trained force branch versus experiment, with mean peak-force errors of 0.22% and 0.55%, respectively. This global agreement does not directly validate the local FE/GNN stress field or specimen-specific fracture timing.
2. The DIC screenshot-processing pipeline converts 25,645 source images from eight specimens into 2509 normalized pseudo-DIC fields and maps the experimental spatial information to the front FE mesh using a valid mask and confidence map. With the base GNN and force branch frozen, the DIC spatial shape loss on the independent L-7 and L-8 test specimens improves by 3.21%, with a mean absolute stress correction of 0.232 MPa. The screenshot-derived information therefore adjusts the local spatial distribution conservatively without materially changing the base stress magnitude or global mechanical evolution. A 72-case synthetic screenshot benchmark supports recovery of relative spatial morphology under the tested degradations. The 3.21% value is a spatial-consistency improvement, not a measure of physical stress-accuracy improvement.
3. Simulation-domain ablations further show that explicit loading/material conditioning is important in the present architecture. Removing FiLM increases the stress RMSE from 4.60 to 75.57 MPa, while reducing the message-passing depth to four layers increases the RMSE to 5.11 MPa. Guide-force regularization has a more modest effect, whereas omitting Stage II force-branch fine-tuning increases the dense force-curve RMSE from 59.70 to 67.85 N, confirming the functional decoupling between field prediction and curve optimization. A node-wise conditional MLP without graph message passing still achieves R^2^ = 0.9932 and RMSE = 9.56 MPa, confirming that the fixed-topology problem is comparatively structured; the GNN reduces overall stress RMSE by 51.9% relative to this baseline. The no-FiLM result should not be interpreted as proof that FiLM is universally optimal.
4. DIC adapter ablations show that experimental spatial supervision and conservative regularization are both necessary. Without DIC spatial supervision, the shape-loss improvement is −0.09%. Removing identity preservation increases the apparent improvement to 58.14%, but also increases the mean correction to 9.021 MPa and the residual TV to 0.019839, indicating clear over-correction. Removing low-pass pooling slightly increases the shape improvement to 3.59% but also increases residual TV. The advantage of the complete adapter therefore lies in balancing experimental consistency, correction amplitude, and spatial smoothness rather than minimizing a single loss metric.
5. Increasing the number of training specimens from one to five raises the independent-test shape-loss improvement from 2.36% to 3.31% and markedly reduces variability across specimen combinations. The main value of additional physically independent specimens is therefore not only improved mean spatial regularization performance, but also reduced sensitivity to the speckle quality and image characteristics of any individual specimen.

The conclusions of this study are restricted to the current parameter space, which uses a fixed geometry, a prescribed central weakened region, and elastic modulus as the primary varying material parameter. The pseudo-DIC fields provide only relative spatial information within each frame and cannot serve as experimental stress or strain ground truth with absolute physical units. The reported results should therefore be interpreted as experiment-informed spatial regularization of the predicted simulation-derived stress field rather than direct inversion of true experimental stress. Future work should introduce joint variations in yield, hardening, and damage parameters and incorporate frame-synchronized numerical DIC displacement/strain tensors and directly synchronized frame-level machine records, and additional geometries and defect locations. Metal additive manufacturing has considerable potential in aerospace applications, but the formation, performance implications, and mitigation of pore defects remain major barriers to broader engineering deployment [36,37,38]. Additive manufacturing of high-strength aluminum alloys also faces substantial challenges, while hybrid additive-manufacturing strategies provide promising routes for improving microstructure and mechanical performance [39,40]. Systematic optimization of process parameters and microstructural control remains central to achieving high performance and reliability in AlSi10Mg components [41,42,43]. The new machine force–displacement comparison provides direct global-response evidence for both the FE reference and the trained force branch, but local FE/GNN stress remains unvalidated by quantitative experimental stress or frame-synchronized numerical DIC tensors. The force-branch comparison uses the common nominal material condition rather than specimen-specific inverse-identified parameters, and the exact terminal element deletion time is mesh-sensitive. Future validation should therefore broaden the material parameter space and include additional geometries, defect locations, and frame-synchronized numerical DIC data.

## Figures and Tables

**Table 1:** AlSi10Mg material parameters and specimen/finite-element settings.

Parameter	Baseline Value/Setting	Notes
Elastic modulus E	60 GPa	LHS scaling factor: 0.92–1.08
Poisson ratio ν	0.30	Fixed across all parametric cases
Primary element type	C3D8R	Reduced-integration solid element
Overall mesh size	Approx. 0.5 mm	Shared topology across all cases
First plastic stress point	133.99 MPa	J2 elastoplasticity + multilinear hardening
Damage model	Ductile damage	Approx. 1 mm central weakened band
Total specimen length	80.94 mm	Full three-dimensional tensile specimen
Gauge-section width × length	6 mm × 32 mm	Grip width: 10 mm
Thickness	2 mm	-
Maximum axial displacement	6.0 mm	Abaqus/Explicit
Analysis time	0.06 s	Explicit loading step
Plasticity/hardening	J2 plasticity; 133.99 MPa yield point to 415 MPa at ε_p_ = 0.38	Tabulated multilinear isotropic hardening
Damage initiation (bulk/weak band)	0.35/0.14	Ductile criterion; triaxiality 0.33; strain rate 0
Damage evolution (bulk/weak band)	0.001/0.15 mm	Displacement-based; element deletion enabled
Weak-band definition	y = −15.47 ± 0.50 mm	Same elastic–plastic law as bulk; damage parameters differ
Loading/mass scaling	6.0 mm in 0.06 s; target Δt = 1 × 10^−6^ s	Smooth-step loading; semi-automatic mass scaling

**Table 2:** Independent test performance in the simulation domain.

Metric	Result
Nodal stress R^2^	0.9984
Nodal stress RMSE/MAE	4.60/1.80 MPa
Mean frame-wise R^2^/relative L2 error	0.9822/3.95%
Nodal displacement RMSE	0.0309 mm
Dense force-curve R^2^/RMSE	0.9957/59.70 N
Single-state full-field + force query	29.88 ± 0.92 ms
201-point force-curve query	3.07 ± 0.07 ms
Node-wise conditional MLP R^2^/RMSE/MAE	0.9932/9.56/4.95 MPa
MLP hotspot/late/damage-zone RMSE	15.77/18.52/42.78 MPa

**Table 3:** Ablation results for the weakly supervised DIC adapter.

Adapter Configuration	Improvement in Spatial Shape Loss/%	Mean |Δσ|/MPa	Normalized Residual TV
Full adapter	3.35 ± 0.07	0.224	0.000667
w/o DIC spatial supervision	−0.09	0.000	0.000000
w/o identity preservation	58.14	9.021	0.019839
w/o gradient consistency	3.26	0.217	0.000645
w/o low-pass pooling	3.59	0.229	0.000810

## Data Availability

The original data presented in the study are openly available in GitHub at https://github.com/Stuartor/alsi10mg-gnn (accessed on 15 September 2026).
